# Novel Insights Into Biarticular Muscle Actions Gained From High-Density Electromyogram

**DOI:** 10.1249/JES.0000000000000254

**Published:** 2021-05-04

**Authors:** Kohei Watanabe, Taian Martins Vieira, Alessio Gallina, Motoki Kouzaki, Toshio Moritani

**Affiliations:** 1 Laboratory of Neuromuscular Biomechanics, School of Health and Sport Sciences, Chukyo University, Nagoya, Japan; 2 Laboratory for Engineering of the Neuromuscular System, Electronics and Telecommunication Department, Politecnico di Torino; 3 PoliToBIOMed Lab, Politecnico di Torino, Torino, Italy; 4 Centre of Precision Rehabilitation for Spinal Pain (CPR Spine), School of Sport, Exercise and Rehabilitation Sciences, College of Life and Environmental Sciences, University of Birmingham, Birmingham, UK; 5 Laboratory of Neurophysiology, Graduate School of Human and Environmental Studies, Kyoto University; 6 Faculty of Sociology, Kyoto Sangyo University, Kyoto, Japan

**Keywords:** neuromuscular compartment, two-joint muscles, multichannel surface EMG, biarticular muscle actions, regional activation

## Abstract

Biarticular muscles have traditionally been considered to exhibit homogeneous neuromuscular activation. The regional activation of biarticular muscles, as revealed from high-density surface electromyograms, seems however to discredit this notion. We thus hypothesize the regional activation of biarticular muscles may contribute to different actions about the joints they span. We then discuss the mechanistic basis and methodological implications underpinning our hypothesis.

## Abstract 2

Human biarticular muscles are regionally activated for different functional roles, and this should be considered when recording surface electromyography.

**Key points**

- Biarticular muscles have traditionally been considered to show a well-defined action and behave as a single actuator, and this is now being questioned.
- Our recent studies using high-density surface electromyography and intramuscular electromyograms suggest that, in humans, different regions within biarticular muscles, such as rectus femoris and medial gastrocnemius muscles, may have different actions.
- We provide guidance on how to conduct surface electromyography of the biarticular muscles to minimize misinterpretation of the findings because of their region-specific neuromuscular activation.

## INTRODUCTION

Biarticular muscle action has been widely investigated in the research areas of human movements. Previous studies from the group of van Ingen Schenau (1,2) support the hypothesis that biarticular muscles play an important role in transferring power from proximal to distal joints. For example, biarticular muscles in the thigh have a unique role in controlling the distribution of the net moments about the hip and knee joints (1). On the other hand, activation of biarticular muscle also contributes to the production of torque in the opposite direction when the torque demand is imposed on either of the two joints they span (3). This condition is considered to be metabolically inefficient and has been called Lombard's paradox (3,4).

The clinical relevance of inappropriate or impaired activation of rectus femoris (RF) is substantiated by the emergence of a pathological gait profile in persons with neurological disorders (5–7). Moreover, it is well known that strain injury during athletic events frequently occurs in biarticular, lower-extremity muscles such as the RF, hamstring, and gastrocnemius muscles (8–13).

Biarticular muscles are often considered to show well-defined actions based on their anatomy and to function as single actuators in experimental and modeling studies (14,15). However, this concept may be questionable. For example, animal studies demonstrated that different regions of biarticular muscles, innervated by different nerve branches, may contribute to different joint actions. In the cat biceps femoris muscle, which crosses the knee and hip joints and is innervated by multiple motor nerve branches, the caudal portion of the muscle contributes to knee flexion, whereas its rostral part contributes to hip extension during locomotion (16). Similarly, human cadaveric studies also assumed this heterogeneous, functional organization within some biarticular muscles, based on their anatomical properties (17). In addition, nonuniform activation within the human RF and medial gastrocnemius (MG) muscles was confirmed by an *in vivo* high spatial resolution methodology, that is, muscle functional magnetic resonance imaging (mfMRI) (18–20).

**Figure 1:**
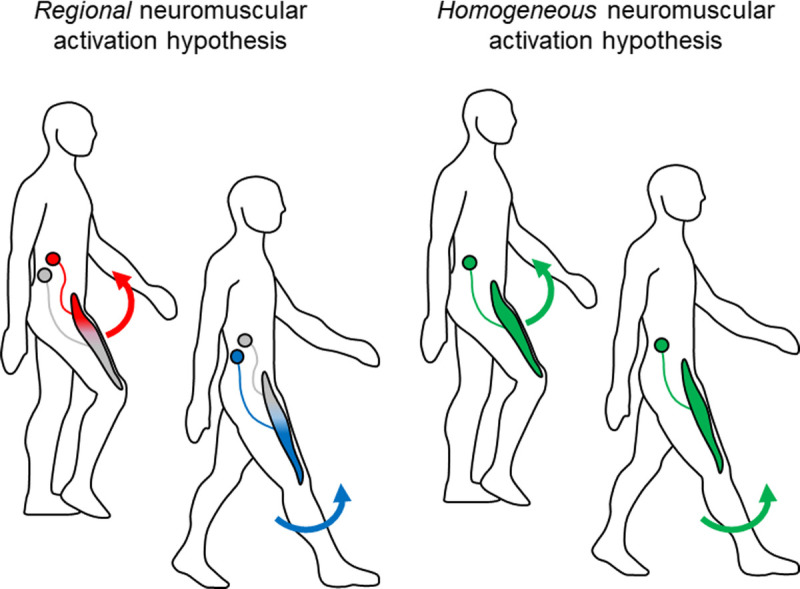
Schematic images of hypotheses for regional and homogeneous neuromuscular regulation within biarticular muscles during human movements.

As a consequence of these findings, presumably, biarticular muscles in humans may be controlled regionally, as if a single biarticular muscle was composed of multiple actuators. Recently, this possibility has been tested by recording the spatial distribution of the amplitude and frequency of surface electromyograms (EMGs) using the high-density surface EMG technique (21–24). The aim of our article was to present and discuss recent evidence supporting the hypothesis that biarticular muscles may be activated regionally, contributing to different actions about the joints they span as suggested by the scheme shown in Figure 1.

### Region-Specific Functional Roles of Human Biarticular Muscles

The torque produced by a muscle about a joint is defined by its proximal and distal attachments to the skeleton. However, individual motor units are not uniformly activated when the muscle contributes to multiple joint actions (25–27). In addition, some human skeletal muscles, such as the biceps brachii, extensor carpi radialis longus, RF, and lateral gastrocnemius, are innervated by multiple motor nerves or nerve branches, leading to subdivisions referred to as neuromuscular compartments (28–31). In this section, we review the recent findings regarding the region-specific functional roles from studies using high-density surface EMG in three human biarticular muscles: RF, MG, and biceps femoris long head (BF) muscles.

#### Region-specific functional roles of human RF muscle

For the human RF muscle, superficial-proximal regions and deep middle–to–distal regions are innervated by different nerve branches and are inserted into different parts of the pelvic bone (17,31,32). Hasserman *et al.* (17) suggested the possibility of independent actions of two muscle-tendon units within the RF muscle from their human cadaver–based anatomical study. In line with this, hip flexion and knee extension forces differed when proximal, middle, and distal regions of the RF muscle were activated with intramuscular electrical stimulation (33). This region specificity was also observed after repetitive isokinetic knee extension exercise, as mfMRI revealed greater changes in metabolic responses (T2) in the distal regions of the RF muscle when compared with proximal regions (18). Selective hypertrophy in distal regions of the RF muscle after resistance training intervention of knee extension exercises (34,35) may also support the preferential activation of distal regions of the RF muscle for knee extension. Overall, the evidence suggests that RF neuromuscular organization facilitates the selective control of force at the knee and hip joints.

**Figure 2:**
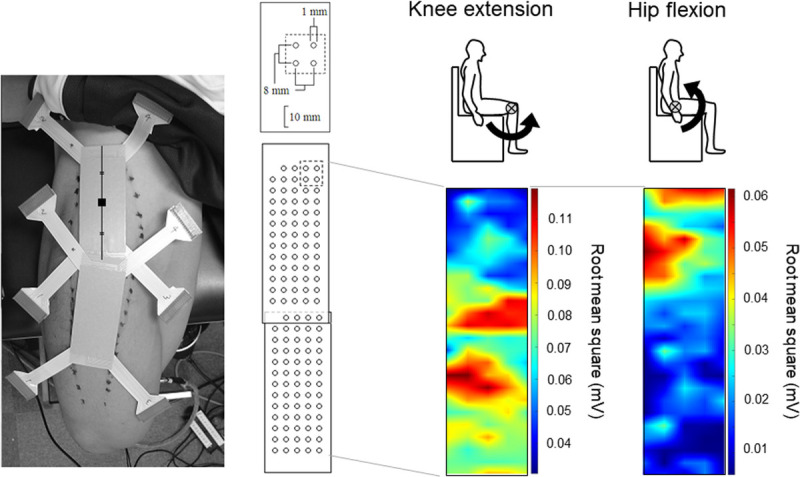
Root-mean-square of surface electromyography in RF muscle during isometric knee extension and hip flexion at 80% of maximal voluntary contraction, as shown in color maps in a representative participant. Surface electromyography was recorded using 128 two-dimensionally arranged electrodes. In these color maps, root-mean-square was normalized by peak amplitude during maximal voluntary contractions across knee extension or hip flexion for each channel. Adapted with permission from Elsevier from Watanabe K, et al (23). Copyright © 2012 Elsevier. All permission requests for this image should be made to the copyright holder.

Recently, we used high-density surface EMG to expand this concept of functional organization within the RF muscle, investigating whether these regional inhomogeneities were present during voluntary isometric knee extension and hip flexion contractions (23). As the contraction level increased, normalized surface EMG amplitudes in proximal and distal regions were preferentially increased during hip flexion and knee extension, respectively (Fig. 2). During hip flexion, normalized surface EMG amplitude was greater in proximal regions than in middle and distal regions (Fig. 2). During knee extension, the opposite pattern was observed. This phenomenon was also confirmed by intramuscular (23) and conventional surface EMG (36) techniques. From these findings, we concluded that proximal and middle/distal regions of the RF muscle preferentially contribute to hip flexion and knee extension and that this region-specific neuromuscular activation is in line with that hypothesized based on anatomical studies (17,31,32).

**Figure 3:**
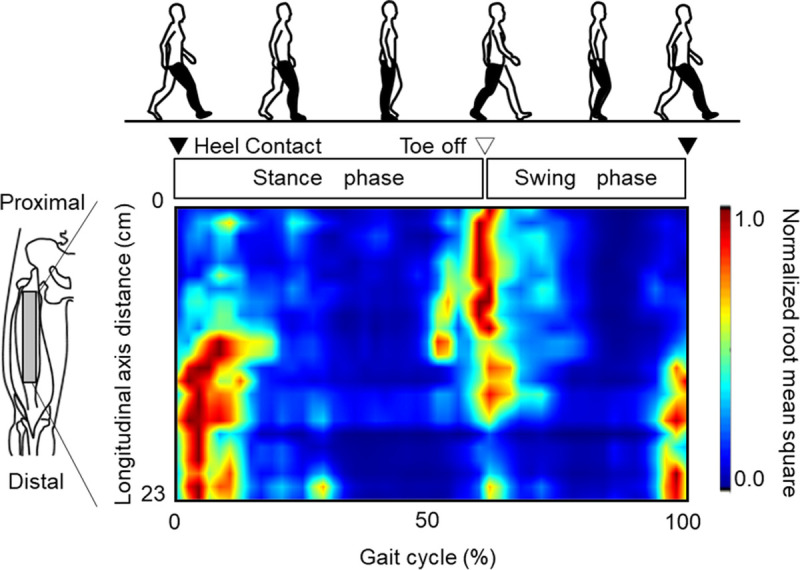
Normalized root-mean-square of surface electromyography in RF muscle during a gait cycle of treadmill walking, as shown in color maps in a representative participant. Surface electromyography was recorded using 48 electrodes along proximal to distal sites. Adapted with permission from Elsevier from Watanabe K, et al (24). Copyright © 2014 Elsevier. All permission requests for this image should be made to the copyright holder.

We further performed high-density EMG of the RF muscle during isometric contractions at various joint angles (37) and dynamic multiple joint movements such as walking (24), climbing stairs (38), and leg pedaling (39) to verify whether region-specific neuromuscular activation may generalize more practical conditions. For example, larger high-density surface EMG amplitude values were localized around the middle regions during the swing-to-stance transition and moved to proximal regions during the stance-to-swing transition while walking (24) (Fig. 3). In addition, greater high-density surface EMG amplitude values were observed in middle/distal regions during the downstroke phase and moved to proximal regions during the upstroke phase of leg pedaling (39). The swing phase during walking and upstroke phase during leg pedaling require hip flexion moment, whereas stance and downstroke phases require knee extension moment (40,41). Therefore, these findings suggest that, regardless of the testing condition, the proximal and distal RF muscle regions preferentially contribute to hip flexion and knee extension moments, respectively.

For the simple bipennate muscles that have two attachments at proximal and distal ends, the forces generated by individual muscle fibers that do not span the entire length of a muscle can be transmitted to the attachment sites on the skeleton or other tissues (42). Regarding the RF muscle, the cadaveric studies revealed that the proximal tendon has two components, which are named as direct and indirect heads (17,32,43). The tendon of the direct head arises from the anterior inferior iliac spine and forms the superficial anterior tendon that covers the ventral aspect of the proximal third of the muscle's length. Conversely, the tendon of the indirect head arises from the superior acetabular ridge and travels along with the tendon of the straight head (17). In addition, direct and indirect heads create independent muscle-tendon units (17,32,43). Based on these anatomical studies, we can assume that proximal and middle/distal regions can independently produce forces to different parts of the skeleton such as the anterior inferior iliac spine and superior acetabular ridge. Because the origin of the direct head is in a more anterior position relative to the femur bone, muscle fibers of the direct head (proximal regions) more favorably create hip flexion moment because of the longer moment arm. In the RF muscle, Hagio *et al.* (33) demonstrated that selective intramuscular electrical stimulation in the proximal region induces more hip flexion moment than in middle or distal regions. In addition, von Laßberg *et al.* (45) recently described the “interfiber-to-tendon interaction model” for proximal to distal regions of the RF muscle and suggested that this model can explain the findings of our recent studies (24,37,39,44). In this model, elastic energy could be buffered and then used to contribute to hip flexion or knee extension according to differential sequences of proximodistal, distoproximal RF activation (45). Thus, we consider that forces generated by muscle fibers in proximal or middle/distal regions of the RF muscle may preferentially contribute to specific joint moment.

#### Region-specific functional roles of human MG muscle

Possibilities of region-specific functional roles were also observed in the MG and RF muscles in humans. The local activation within the human gastrocnemius muscles was investigated using intramuscular EMG (46) and mfMRI (20,47). Kinugasa *et al.* (19) found that increases in metabolic responses (T2) on mfMRI were greater in distal regions when compared with proximal regions within the MG muscle. Task-dependent regional neuromuscular activation within the lateral gastrocnemius muscle was reported by Wolf *et al.* (46) using intramuscular EMG. Vieira *et al.* (22) tested whether muscle fibers innervated by a motor neuron are regionally localized or widely extend along the human MG muscle using intramuscular and high-density surface EMG. From the amplitude distribution of action potentials of single motor units detected along the skin, they advanced the possibility that the muscle fibers of single motor units (*i.e.*, the muscle units) are localized along the muscle and that MG muscle units recruited during quiet standing were predominantly detected in the distal muscle region. The study of Hodson-Tole *et al.* (48) supported this task-dependent preferential recruitment of distal regions of MG muscle during voluntary contractions. Region-specific neuromuscular regulation along the medial-lateral axis within the MG muscle was also revealed in response to lateral perturbation during unilateral standing (49), suggesting that this regional activation is associated with MG regional contributions to inversion and eversion moments (50). These studies suggest that the MG muscle is regionally recruited for specific tasks, rather than being regulated as a single actuator. It may be difficult to transmit forces from muscle fibers in different regions in varying directions or with differing joint torque. Although the line of action of the MG muscle should be along the line between the origin and skeletal insertion, that is, the calcaneus and medial condyle, the attachments of individual muscle fibers to the tendon tissues connected to the calcaneus and medial condyle are variable along the proximal to distal axis of that muscle. For example, the muscle fibers of distal regions are closer to the Achilles tendon, which is connected to the calcaneus. In addition, because the muscle fibers in distal regions are less pennate than those in proximal regions along the MG muscle (cf. Figs. 1D, 4B in Shin *et al.* [51]), it was considered that the muscle fibers in distal regions have greater mechanical advantages to generate ankle plantar flexion (48).

#### Region-specific functional roles of human biceps femoris muscle

In addition to RF and MG muscles, region-specific neuromuscular activation along the proximal to distal axis of BF and semitendinosus (ST) muscles can be assumed because of their innervation patterns. The BF and ST muscles are innervated by two motor nerve branches that are attached into different sites along the proximal-distal axis of the muscle (52) similar to the RF muscle (31). Recent studies reported regional activation along the BF and ST muscles and its task dependency on common and injury prevention hamstring exercises (53,54). For example, distal regions showed lower and higher neuromuscular activation when compared with proximal and middle regions in ST and BF muscles, respectively, during the Nordic hamstring exercise but not during the stiff-leg deadlift (54). They also showed regional neuromuscular activation along ST and BF muscles during running, although with marked interindividual differences (55). On the other hand, there were no significant differences in neuromuscular activations among proximal to distal regions of the BF muscle during isometric hip extension and knee flexion in our previous study (56). From these findings, although BF and ST muscles could have regional neuromuscular activation along proximal to distal regions, a consistent pattern of region-specific neuromuscular activation such as in RF and MG muscles has not yet been identified.

### Physiological Basis and Roles Underpinning the Region-Specific Excitation of Human Biarticular Muscles

As reported in previous studies (25–27), motor units may contribute to different functions within individual muscles. We thus assume that the regional neuromuscular activation along the RF and MG muscles in different tasks can be explained by a differential modulation of presynaptic activity of motor neurons serving different muscle regions. To our knowledge, this possibility has been first advocated by Desmedt and Godeaux (1981). These authors observed that motor units in the first dorsal interosseus muscle may be selectively recruited according to the force direction, suggesting pools of motor units with specific function receive differential, presynaptic inputs (cf. their Fig. 2) (57). In agreement with this view, we found that patellar tendon reflex responses of proximal RF different when the flexion moment about the knee joint was varied (58). In addition, we showed that cutaneous electrical stimulation at femoral and posterior tibial nerves elicits region-specific action potentials along the RF and MG muscles (48,59). These findings suggest that motor units with territories located in proximal and distal regions of the RF and MG muscles may be independently controlled, presumably according to their functional roles at the spinal cord level or upstream of motor neuron.

In this review, we hypothesize that regional activation of biarticular muscles enables them to contribute to different actions about the joints they span. The reason we phrased the relevance of regional excitation rather vaguely is that, in addition to contributing to the generation of muscle force in different directions and thus to different movements, the regional excitation of biarticular muscles could serve other purposes. Indeed, the local excitation of distinct regions has been consistently reported under different, fatiguing conditions and for different muscles (60–63). As documented by our group (21,64), this evidence for region-specific adjustments in myoelectric activity during fatiguing contractions also applies to the biarticular RF and MG muscles, supporting our hypothesis that muscle activation is modulated regionally in human biarticular muscles.

**Figure 4:**
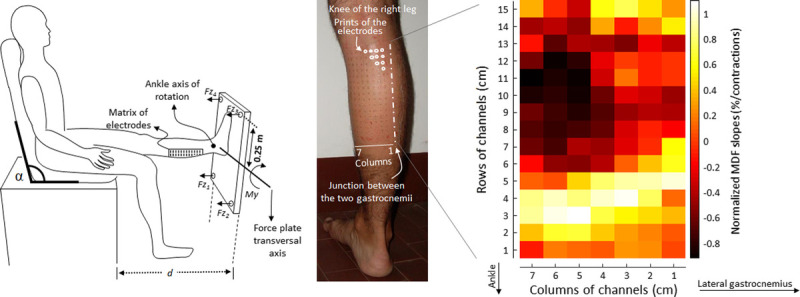
Normalized median frequency (MDF) slope of surface electromyography in MG muscle during isometric fatiguing contraction. Surface electromyography was recorded using 128 two-dimensionally arranged electrodes. Adapted with permission from Elsevier from Gallina A, et al (21). Copyright © 2011 Elsevier. All permission requests for this image should be made to the copyright holder.

During isometric sustained contractions, nonuniform neuromuscular activation in the course of fatiguing conditions has been reported in human skeletal muscles. This phenomenon has been partly explained by the substitution/rotation of different motor units, whose fibers are heterogeneously distributed within a muscle (65,66), and is assumed to occur after changes in recruitment thresholds in motor units and decreases in motor unit firing rate induced by inhibitory feedback from group III and IV afferents (65). In addition to this nonsystematic alteration of neuromuscular activation within a muscle, region-specific neuromuscular fatigue in biarticular muscles was also reported using high-density surface EMG. In the RF muscle, greater decreases in the median frequency of surface EMG were observed in proximal regions in comparison with middle/distal regions during sustained isometric contraction of knee extension at 50% of MVC until the exerted force falls below 95% of the target force (64). Similarly, Gallina *et al.* (21) demonstrated localized adjustments in myoelectric activity during fatiguing contractions in centro-proximal regions of the MG muscle during isometric plantar flexion (Fig. 4). From these studies, we consider that the adjustments in myoelectric activity are observed to take place locally within these muscles. These local adjustments could result from a predominant recruitment of motor units with fibers in these locations or be associated with the location of fibers of the most fatigable motor units within the muscle. In support of this view, Hodson-Tole *et al.* (48) and Vieira *et al.* (67) showed the preferential recruitment of proximal regions of the MG muscle during electrically elicited contraction. Using a similar methodology, we also confirmed that electrical nerve stimulation can preferentially recruit motor units located in proximal regions of the RF muscle (59). According to the consideration that larger motor nerves, innervating muscle fibers that contribute to higher contraction forces, are preferentially activated by nerve stimulation (68,69), the regions that are selectively activated by electrically elicited contraction in these previous studies may be composed of muscle fibers that contribute to higher contraction forces. Thus, we conclude that region-specific adjustments in myoelectric activity during fatiguing contractions within RF and MG muscles can be partly explained by a nonuniform distribution of muscle fiber types within muscles.

The possibility that fibers are grouped within some muscles according to their properties has been further supported by anatomical studies (70–73). For example, Arbanas *et al.* (70) demonstrated a higher percentage of type II muscle fibers in caudal parts compared with cranial parts within the human psoas major muscle. Based on the anatomical properties of the origins and insertions of the muscle fibers and the region-specific muscle-fiber–type composition, they considered that cranial and caudal parts of the psoas major muscle may preferentially contribute to the production of hip flexion as a dynamic function and control of the lower spine posture as a static postural function. RF and MG muscles play the roles of knee extensor and ankle plantar flexor in addition to the roles of hip and knee flexors, respectively. During daily life, knee extensors and ankle plantar flexors help stabilize the body against gravity. Considering that body stabilization demands low and frequent cyclic activation of these postural muscles, activation of muscle fibers that contribute to lower forces for static postural functions may be reasonable. This is supported by our previous studies that revealed preferential activation of middle-distal regions of RF and MG muscles during knee extension and quiet standing (22,23,48) and less adjustments in myoelectric activity during fatiguing contractions in distal than proximal regions during sustained contractions (21,64).

### Region-Specific Functional Roles and Its Applications to Assess Impairments in Human Movements

Biarticular muscles produce a torque about each of the joints they span. For example, when hip flexion joint torque is exerted by the hip flexor muscles, including the RF muscle, knee extension joint torque also is produced by contraction of the RF muscle. During walking, leg swing demands the concurrent flexion of the knee and hip joints to prevent tripping, dragging, and/or falling by contacting the ground with the foot (41,74). Excessive activation of the RF muscle would contribute to prevent knee flexion torque or change the knee joint kinematics, at the cost of producing a hip flexion torque for leg swing. This disadvantage of biarticular muscle actions can be seen in pathological gait patterns. Abnormality in neuromuscular control of the RF muscle causes a stiff-knee gait in patients with stroke, traumatic brain injury, spinal cord injury, cerebral palsy, or multiple sclerosis (5,7,75). During stance-to-swing transition, these patients show excessive RF muscle activation (7,74). This hyperactivity of the RF muscle prevents the knee flexion movement necessary for smooth leg swinging and, thereby, impairs toe clearance. Distal transfer and nerve block of the RF muscle are thus common treatments for a stiff-knee gait (6,30,76). Our previous study demonstrated that selective activation of proximal regions of the RF muscle during the stance-to-swing transition is attenuated in older adults during walking, that is, decrease and tendency to increase in normalized surface EMG amplitude at the proximal and distal regions, respectively, and then distal shift of central locus activation (center of gravity) of surface EMG amplitude in the swing phase (44). We also confirmed that this age-related attenuation of region-specific activation of the RF muscle shows marked interindividual differences (77) and is modulated differently between older adults with and without a fall/tripping history (78). From these findings, for patients with upper motor neuron injury and older adults, neuromuscular regulation of the RF muscle and its regional activation may be key determinants for leg swing movement during walking and complex human movements. For the MG muscle, Dos Anjos *et al.* (79) showed higher neuromuscular activation in distal regions of the muscle in young adults and higher neuromuscular activation from proximal to distal regions in older adults during standing. They suggested that a relatively larger proportion of MG motor units may have been recruited in the older adults.

Regional activation within the biarticular muscles may be also reflected in changes in muscle morphology after aging or inactivity. In the RF muscle, age-related changes in the muscle thickness were not uniform along the muscle (80). In addition, Miokovic *et al.* (81) demonstrated heterogeneous muscle atrophy along the proximal to distal axis after a 60-d bed rest in RF, MG, and BF muscles. These regional changes in morphology along the muscle would also have implications when investigating region-specific neuromuscular activation along the muscle after age- or inactivity-related changes in the neuromuscular system.

It has been recognized that biarticular muscles contribute to transferring power from proximal to distal joints (1,2) and partly act as inefficient components (3,4) when performing multiple joint movements. Although further studies are needed to clarify how regional neuromuscular activation contributes to these well-known functional roles of biarticular muscle, our previous studies showed that regional neuromuscular activation along the RF and MG muscles adapt to age-related changes in human movements (44,78,79). This indirectly suggests that regional neuromuscular activation could contribute to the optimization of the transfer of power from proximal to distal joints (1,2) and the minimization of the inefficient joint moments (3,4).

### Methodological Implication for Assessing the Region-Specific Functional Roles Within Biarticular Muscles

When using conventional bipolar electromyography, activation of the RF muscle during gait in healthy individuals is characterized by two main bursts, around the swing-to-stance transition and the stance-to-swing transition. We confirmed in our previous study, using multiple electrodes along the proximal to distal sites of the RF muscle, that this often-termed “normal” pattern (82–84) can only be detected in a very limited area around the middle regions (24,44). This limited area reflects activations of both proximal and distal regions, which preferentially contribute to hip flexion and knee extension. When detecting a single EMG signal, from either a more proximal or distal region, our results (24,44) suggest that different conclusions may be made depending on the state of RF muscle activation; one may miss the EMG bursts characterizing the swing-to-stance and stance-to-swing transitions and equivocally judge the EMG pattern as pathological. Similarly, conventional bipolar EMG was shown to be less sensitive than high-density EMG in the detection of adjustments in myoelectric activity during fatiguing contractions: the decrease in the median EMG frequency over time was larger and more consistent when estimated from specific areas within the MG muscle, compared with estimates from a standardized group of channels that simulated the size and location of conventional bipolar electrodes (21). As for the RF muscle, detecting a single EMG signal may lead to a spurious difference in adjustments in myoelectric activity during fatiguing contractions (64). Acknowledging the phenomenon of regional neuromuscular activation within biarticular muscles and considering this knowledge when devising a study may be mandatory whenever inferences are to be drawn from surface EMG.

**Figure 5:**
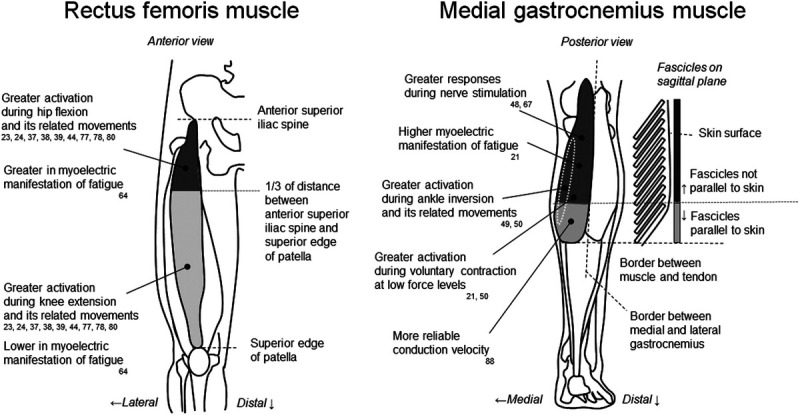
Anatomical maps of region-specific activation within RF and MG muscles based on the results of previous studies using high-density surface electromyography.

Regarding practical guidelines, in an attempt to aid those without access to the high-density technology, we propose a set of recommendations concerning the use of bipolar EMG. These recommendations are specifically for RF and MG muscles and based on the region-specific activation reported in previous studies using high-density surface EMG (Fig. 5). For the RF muscle, we recommend using two surface EMG electrode pairs, over the proximal and middle-distal regions, to assess RF muscle contributions to hip flexion and knee extension. Based on previous studies (23,36,58,85,86), surface electrode pairs at 20% and 50% or more distal sites along a line between the anterior superior iliac spine and superior edge of the patella from the proximal site would lead to neuromuscular activation of the RF muscle to mainly act as a hip flexor or knee extensor. Under fatiguing conditions, proximal electrodes would be expected to show greater adjustments in myoelectric activity during fatiguing contractions than middle-distal electrodes.

For the MG muscle, the largest decrease in median frequency during intermittent contractions was observed for electrodes positioned 66–131 mm proximal to the junction with Achilles tendon and 38–53 mm medial to the junction with the lateral gastrocnemius (21). Based on anthropometric data of subjects tested in our previous study, this absolute reference would roughly correspond to the central MG region (21), from the distal extremity of the superficial aponeurosis to popliteal fossa. Bipolar electrodes placed in this region may provide the most sensitive estimates of adjustments in myoelectric activity during fatiguing contractions. These recommended electrode locations for RF and MG muscles are likely to improve the representation of surface EMG variables collected from the target muscle. This would result in improved characterization of the activation of the muscles while minimizing misunderstanding of the detected EMG signals because of their region-specific neuromuscular activations. We also need to note here that whenever placing more than a single pair of electrodes on a muscle, researchers should ensure that these electrode pairs are located on muscle regions with similar architectures and subcutaneous tissues (cf. Fig. 6 in Vieira and Botter (87)). For example, the arrangement between electrode pairs on the skin surface and muscle fibers/fascicles is markedly different along the MG muscle. Electrode pairs are not parallel to fascicles at proximal to middle sites but are parallel to fascicles at distal sites (right panel in Fig. 5), and these differences would also lead to variations in surface EMG responses. When this condition cannot be ensured, the regional differences in surface EMG variables may lead to the inability to gain information on region-specific functional roles (88).

## CONCLUSION

Recent studies using the high-density surface EMG technique demonstrated region-specific activations that can be interpreted as regional differences in functional roles within representative biarticular muscles, such as RF and MG muscles. These findings support our hypothesis that human biarticular muscles are regionally regulated to achieve different functional roles. This knowledge and its application to practical surface EMG recording may help advance understanding of the role of biarticular muscles in human movement.

## References

[1] Jacobs R van Ingen Schenau GJ. Control of an external force in leg extensions in humans. *J. Physiol*. 1992; 457:611–26. 1297846 10.1113/jphysiol.1992.sp019397 PMC1175750

[2] van Ingen Schenau GJ Dorssers WM Welter TG Beelen A de Groot G Jacobs R. The control of mono-articular muscles in multijoint leg extensions in man. *J. Physiol*. 1995; 484(Pt 1):247–54. 7602524 10.1113/jphysiol.1995.sp020662 PMC1157936

[3] Gregor RJ Cavanagh PR LaFortune M. Knee flexor moments during propulsion in cycling-a creative solution to Lombard's paradox. *J. Biomech*. 1985; 18(5):307–16. 4008501 10.1016/0021-9290(85)90286-6

[4] Lombard WP. The action of two-joint muscle. *Am. Phys. Educ. Rev*. 1903; 8:141–5.

[5] Annaswamy TM Giddings CJ Della Croce U Kerrigan DC. Rectus femoris: its role in normal gait. *Arch. Phys. Med. Rehabil*. 1999; 80(8):930–4. 10453770 10.1016/s0003-9993(99)90085-0

[6] Chantraine F Detrembleur C Lejeune TM. Effect of the rectus femoris motor branch block on post-stroke stiff-legged gait. *Acta Neurol. Belg*. 2005; 105(3):171–7. 16255155

[7] Reinbolt JA Fox MD Arnold AS Ounpuu S Delp SL. Importance of preswing rectus femoris activity in stiff-knee gait. *J. Biomech*. 2008; 41(11):2362–9. 18617180 10.1016/j.jbiomech.2008.05.030 PMC5507200

[8] Delgado GJ Chung CB Lektrakul N, . Tennis leg: clinical US study of 141 patients and anatomic investigation of four cadavers with MR imaging and US. *Radiology*. 2002; 224(1):112–9. 12091669 10.1148/radiol.2241011067

[9] Green B Pizzari T. Calf muscle strain injuries in sport: a systematic review of risk factors for injury. *Br. J. Sports Med*. 2017; 51(16):1189–94. 28259848 10.1136/bjsports-2016-097177

[10] Hughes C 4th Hasselman CT Best TM Martinez S Garrett WE Jr. Incomplete, intrasubstance strain injuries of the rectus femoris muscle. *Am. J. Sports Med*. 1995; 23(4):500–6. 7573664 10.1177/036354659502300422

[11] Irmola T Heikkila JT Orava S Sarimo J. Total proximal tendon avulsion of the rectus femoris muscle. *Scand. J. Med. Sci. Sports*. 2007; 17(4):378–82. 16787444 10.1111/j.1600-0838.2006.00564.x

[12] Garrett WE Jr. Injuries to the muscle-tendon unit. *Instr. Course Lect*. 1988; 37:275–82. 3047251

[13] Woodley S Mercer S. Hamstring strains—where do they occur? *NZ Journl of Physiotherapy*. 2004; 32(1):22–8.

[14] Neptune RR Hull ML. Evaluation of performance criteria for simulation of submaximal steady-state cycling using a forward dynamic model. *J. Biomech. Eng*. 1998; 120(3):334–41. 10412400 10.1115/1.2797999

[15] Delp SL Anderson FC Arnold AS, . OpenSim: open-source software to create and analyze dynamic simulations of movement. *I.E.E.E. Trans. Biomed. Eng*. 2007; 54(11):1940–50. 10.1109/TBME.2007.901024 18018689

[16] Pratt CA Chanaud CM Loeb GE. Functionally complex muscles of the cat hindlimb. IV. Intramuscular distribution of movement command signals and cutaneous reflexes in broad, bifunctional thigh muscles. *Exp. Brain Res*. 1991; 85(2):281–99. 1893981 10.1007/BF00229407

[17] Hasselman CT Best TM Hughes C 4th Martinez S Garrett WE Jr. An explanation for various rectus femoris strain injuries using previously undescribed muscle architecture. *Am. J. Sports Med*. 1995; 23(4):493–9. 7573663 10.1177/036354659502300421

[18] Akima H Takahashi H Kuno S Katsuta S. Coactivation pattern in human quadriceps during isokinetic knee-extension by muscle functional MRI. *Eur. J. Appl. Physiol*. 2004; 91:7–14. 14551776 10.1007/s00421-003-0942-z

[19] Kinugasa R Kawakami Y Fukunaga T. Muscle activation and its distribution within human triceps surae muscles. *J. Appl. Physiol (1985)*. 2005; 99(3):1149–56. 15890750 10.1152/japplphysiol.01160.2004

[20] Segal RL Song AW. Nonuniform activity of human calf muscles during an exercise task. *Arch. Phys. Med. Rehabil*. 2005; 86(10):2013–7. 16213247 10.1016/j.apmr.2005.04.012

[21] Gallina A Merletti R Vieira TM. Are the myoelectric manifestations of fatigue distributed regionally in the human medial gastrocnemius muscle? *J. Electromyogr. Kinesiol*. 2011; 21(6):929–38. 21911301 10.1016/j.jelekin.2011.08.006

[22] Vieira TM Loram ID Muceli S Merletti R Farina D. Postural activation of the human medial gastrocnemius muscle: are the muscle units spatially localised? *J. Physiol*. 2011; 589(Pt 2):431–43. 21115645 10.1113/jphysiol.2010.201806 PMC3043543

[23] Watanabe K Kouzaki M Moritani T. Task-dependent spatial distribution of neural activation pattern in human rectus femoris muscle. *J. Electromyogr. Kinesiol*. 2012; 22(2):251–8. 22153052 10.1016/j.jelekin.2011.11.004

[24] Watanabe K Kouzaki M Moritani T. Regional neuromuscular regulation within human rectus femoris muscle during gait. *J. Biomech*. 2014; 47:3502–8. 25246002 10.1016/j.jbiomech.2014.09.001

[25] Ballantyne BT Kukulka CG Soderberg GL. Motor unit recruitment in human medial gastrocnemius muscle during combined knee flexion and plantarflexion isometric contractions. *Exp. Brain Res*. 1993; 93(3):492–8. 8519338 10.1007/BF00229364

[26] ter Haar Romeny BM van der Gon JJ Gielen CC. Relation between location of a motor unit in the human biceps brachii and its critical firing levels for different tasks. *Exp. Neurol*. 1984; 85(3):631–50. 6468581 10.1016/0014-4886(84)90036-0

[27] van Zuylen EJ Gielen CC Denier van der Gon JJ. Coordination and inhomogeneous activation of human arm muscles during isometric torques. *J. Neurophysiol*. 1988; 60(5):1523–48. 3199172 10.1152/jn.1988.60.5.1523

[28] Segal RL Catlin PA Krauss EW Merick KA Robilotto JB. Anatomical partitioning of three human forearm muscles. *Cells Tissues Organs*. 2002; 170(2–3):183–97. 11731706 10.1159/000046191

[29] Segal RL Wolf SL DeCamp MJ Chopp MT English AW. Anatomical partitioning of three multiarticular human muscles. *Acta Anat (Basel)*. 1991; 142(3):261–6. 1796742 10.1159/000147199

[30] Sung DH Jung JY Kim HD Ha BJ Ko YJ. Motor branch of the rectus femoris: anatomic location for selective motor branch block in stiff-legged gait. *Arch. Phys. Med. Rehabil*. 2003; 84(7):1028–31. 12881829 10.1016/s0003-9993(03)00029-7

[31] Yang D Morris SF. Neurovascular anatomy of the rectus femoris muscle related to functioning muscle transfer. *Plast. Reconstr. Surg*. 1999; 104(1):102–6. 10597681

[32] Gyftopoulos S Rosenberg ZS Schweitzer ME Bordalo-Rodrigues M. Normal anatomy and strains of the deep musculotendinous junction of the proximal rectus femoris: MRI features. *AJR Am. J. Roentgenol*. 2008; 190(3):W182–6. 18287410 10.2214/AJR.07.2947

[33] Hagio S Nagata K Kouzaki M. Region specificity of rectus femoris muscle for force vectors in vivo. *J. Biomech*. 2012; 45(1):179–82. 22030124 10.1016/j.jbiomech.2011.10.012

[34] Matta TT Nascimento FX Fernandes IA Oliveira LF. Heterogeneity of rectus femoris muscle architectural adaptations after two different 14-week resistance training programmes. *Clin. Physiol. Funct. Imaging*. 2015; 35(3):210–5. 24750784 10.1111/cpf.12151

[35] Ema R Wakahara T Miyamoto N Kanehisa H Kawakami Y. Inhomogeneous architectural changes of the quadriceps femoris induced by resistance training. *Eur. J. Appl. Physiol*. 2013; 113(11):2691–703. 23949789 10.1007/s00421-013-2700-1

[36] Miyamoto N Wakahara T Kawakami Y. Task-dependent inhomogeneous muscle activities within the bi-articular human rectus femoris muscle. *PLoS One*. 2012; 7(3):e34269. 22479583 10.1371/journal.pone.0034269 PMC3313973

[37] Watanabe K Kouzaki M Moritani T. Non-uniform surface electromyographic responses to change in joint angle within rectus femoris muscle. *Muscle Nerve*. 2014; 50(5):794–802. 24590732 10.1002/mus.24232

[38] Watanabe K Kouzaki M Moritani T. Effect of aging on regional neuromuscular regulation within human rectus femoris muscle during stair ascent and descent. *Gait Posture*. 2017; 52:26–32. 27855311 10.1016/j.gaitpost.2016.11.011

[39] Watanabe K Kouzaki M Moritani T. Heterogeneous neuromuscular activation within human rectus femoris muscle during pedaling. *Muscle Nerve*. 2015; 52(3):404–11. 25524446 10.1002/mus.24544

[40] Gregor RJ Broker JP Ryan MM. The biomechanics of cycling. *Exerc. Sport Sci. Rev*. 1991; 19:127–69. 1936084

[41] Lay AN Hass CJ Gregor RJ. The effects of sloped surfaces on locomotion: a kinematic and kinetic analysis. *J. Biomech*. 2006; 39(9):1621–8. 15990102 10.1016/j.jbiomech.2005.05.005

[42] Bojsen-Moller J Schwartz S Kalliokoski KK Finni T Magnusson SP. Intermuscular force transmission between human plantarflexor muscles in vivo. *J. Appl. Physiol (1985)*. 2010; 109(6):1608–18. 20884838 10.1152/japplphysiol.01381.2009

[43] Bordalo-Rodrigues M Rosenberg ZS. MR imaging of the proximal rectus femoris musculotendinous unit. *Magn. Reson. Imaging Clin. N. Am*. 2005; 13(4):717–25. 16275579 10.1016/j.mric.2005.08.005

[44] Watanabe K Kouzaki M Moritani T. Regional neuromuscular regulation within human rectus femoris muscle during gait in young and elderly men. *J. Biomech*. 2016; 49(1):19–25. 26610353 10.1016/j.jbiomech.2015.11.010

[45] von Laßberg C Schneid JA Graf D Finger F Rapp W Stutzig N. Longitudinal sequencing in intramuscular coordination: a new hypothesis of dynamic functions in the human rectus femoris muscle. *PLoS One*. 2017; 12(8):e0183204. 28817715 10.1371/journal.pone.0183204 PMC5560678

[46] Wolf SL Segal RL English AW. Task-oriented EMG activity recorded from partitions in human lateral gastrocnemius muscle. *J. Electromyogr. Kinesiol*. 1993; 3(2):87–94. 20870530 10.1016/1050-6411(93)90003-F

[47] Kinugasa R Akima H. Neuromuscular activation of triceps surae using muscle functional MRI and EMG. *Med. Sci. Sports Exerc*. 2005; 37(4):593–8. 15809557 10.1249/01.mss.0000159026.99792.76

[48] Hodson-Tole EF Loram ID Vieira TM. Myoelectric activity along human gastrocnemius medialis: different spatial distributions of postural and electrically elicited surface potentials. *J. Electromyogr. Kinesiol*. 2013; 23(1):43–50. 22967836 10.1016/j.jelekin.2012.08.003 PMC3566583

[49] Cohen JW Gallina A Ivanova TD Vieira T McAndrew DJ Garland SJ. Regional modulation of the ankle plantarflexor muscles associated with standing external perturbations across different directions. *Exp. Brain Res*. 2020; 238(1):39–50. 31760455 10.1007/s00221-019-05696-8

[50] Vieira TM Minetto MA Hodson-Tole EF Botter A. How much does the human medial gastrocnemius muscle contribute to ankle torques outside the sagittal plane? *Hum. Mov. Sci*. 2013; 32(4):753–67. 23992638 10.1016/j.humov.2013.03.003 PMC3791398

[51] Shin DD Hodgson JA Edgerton VR Sinha S. In vivo intramuscular fascicle-aponeuroses dynamics of the human medial gastrocnemius during plantarflexion and dorsiflexion of the foot. *J. Appl. Physiol (1985)*. 2009; 107(4):1276–84. 19608924 10.1152/japplphysiol.91598.2008 PMC2763833

[52] Seidel PM Seidel GK Gans BM Dijkers M. Precise localization of the motor nerve branches to the hamstring muscles: an aid to the conduct of neurolytic procedures. *Arch. Phys. Med. Rehabil*. 1996; 77(11):1157–60. 8931528 10.1016/s0003-9993(96)90140-9

[53] Hegyi A Csala D Peter A Finni T Cronin NJ. High-density electromyography activity in various hamstring exercises. *Scand. J. Med. Sci. Sports*. 2019; 29(1):34–43. 30230042 10.1111/sms.13303

[54] Hegyi A Peter A Finni T Cronin NJ. Region-dependent hamstrings activity in Nordic hamstring exercise and stiff-leg deadlift defined with high-density electromyography. *Scand. J. Med. Sci. Sports*. 2018; 28(3):992–1000. 29143379 10.1111/sms.13016

[55] Hegyi A Goncalves BAM Finni T Cronin NJ. Individual region- and muscle-specific hamstring activity at different running speeds. *Med. Sci. Sports Exerc*. 2019; 51(11):2274–85. 31634294 10.1249/MSS.0000000000002060

[56] Watanabe K Kouzaki M Moritani T. Effect of electrode location on task-dependent electromyography responses within the human biceps femoris muscle. *J. Appl. Biomech*. 2016; 32(1):97–100. 26252727 10.1123/jab.2015-0017

[57] Desnedt HE Gidaux E. Spinal motoneuron recruitment in man: rank deordering with direction but not with speed of voluntary movement. *Science*. 1981; 214(4523):933–6. 7302570 10.1126/science.7302570

[58] Watanabe K. Region-specific modulation of tendon reflex along human rectus femoris muscle. *Hum. Mov. Sci*. 2018; 58:224–30. 29486429 10.1016/j.humov.2018.02.013

[59] Watanabe K Kouzaki M Ando R Akima H Moritani T. Non-uniform recruitment along human rectus femoris muscle during transcutaneous electrical nerve stimulation. *Eur. J. Appl. Physiol*. 2015; 115(10):2159–65. 26059495 10.1007/s00421-015-3196-7

[60] Falla D Farina D Graven-Nielsen T. Spatial dependency of trapezius muscle activity during repetitive shoulder flexion. *J. Electromyogr. Kinesiol*. 2007; 17(3):299–306. 16740396 10.1016/j.jelekin.2006.03.005

[61] Farina D Leclerc F Arendt-Nielsen L Buttelli O Madeleine P. The change in spatial distribution of upper trapezius muscle activity is correlated to contraction duration. *J. Electromyogr. Kinesiol*. 2008; 18(1):16–25. 17049273 10.1016/j.jelekin.2006.08.005

[62] Holtermann A Gronlund C Karlsson JS Roeleveld K. Differential activation of regions within the biceps brachii muscle during fatigue. *Acta physiologica (Oxford, England)*. 2008; 192(4):559–67. 10.1111/j.1748-1716.2007.01775.x 18005216

[63] Tucker K Falla D Graven-Nielsen T Farina D. Electromyographic mapping of the erector spinae muscle with varying load and during sustained contraction. *J. Electromyogr. Kinesiol*. 2009; 19(3):373–9. 18061480 10.1016/j.jelekin.2007.10.003

[64] Watanabe K Kouzaki M Moritani T. Region-specific myoelectric manifestations of fatigue in human rectus femoris muscle. *Muscle Nerve*. 2013; 48(2):226–34. 23733312 10.1002/mus.23739

[65] Bawa P Murnaghan C. Motor unit rotation in a variety of human muscles. *J. Neurophysiol*. 2009; 102(4):2265–72. 19657086 10.1152/jn.00278.2009

[66] Westgaard RH de Luca CJ. Motor unit substitution in long-duration contractions of the human trapezius muscle. *J. Neurophysiol*. 1999; 82(1):501–4. 10400978 10.1152/jn.1999.82.1.501

[67] Vieira TM Botter A Minetto MA Hodson-Tole EF. Spatial variation of compound muscle action potentials across human gastrocnemius medialis. *J. Neurophysiol*. 2015; 114(3):1617–27. 26156382 10.1152/jn.00221.2015 PMC4563026

[68] Gregory CM Bickel CS. Recruitment patterns in human skeletal muscle during electrical stimulation. *Phys. Ther*. 2005; 85(4):358–64. 15794706

[69] Hennings K Kamavuako EN Farina D. The recruitment order of electrically activated motor neurons investigated with a novel collision technique. *Clin. Neurophysiol*. 2007; 118(2):283–91. 17174598 10.1016/j.clinph.2006.10.017

[70] Arbanas J Klasan GS Nikolic M Jerkovic R Miljanovic I Malnar D. Fibre type composition of the human psoas major muscle with regard to the level of its origin. *J. Anat*. 2009; 215(6):636–41. 19930517 10.1111/j.1469-7580.2009.01155.x PMC2796786

[71] Korfage JA Van Eijden TM. Regional differences in fibre type composition in the human temporalis muscle. *J. Anat*. 1999; 194(Pt 3):355–62. 10386773 10.1046/j.1469-7580.1999.19430355.x PMC1467935

[72] Lexell J Taylor CC. Variability in muscle fibre areas in whole human quadriceps muscle: effects of increasing age. *J. Anat*. 1991; 174:239–49. 2032938 PMC1256058

[73] Lindman R Eriksson A Thornell LE. Fiber type composition of the human male trapezius muscle: enzyme-histochemical characteristics. *Am. J. Anat*. 1990; 189(3):236–44. 2148051 10.1002/aja.1001890306

[74] Riley PO Kerrigan DC. Torque action of two-joint muscles in the swing period of stiff-legged gait: a forward dynamic model analysis. *J. Biomech*. 1998; 31(9):835–40. 9802784 10.1016/s0021-9290(98)00107-9

[75] Kerrigan DC Gronley J Perry J. Stiff-legged gait in spastic paresis. A study of quadriceps and hamstrings muscle activity. *Am. J. Phys. Med. Rehabil*. 1991; 70(6):294–300. 1741998

[76] Sung DH Bang HJ. Motor branch block of the rectus femoris: its effectiveness in stiff-legged gait in spastic paresis. *Arch. Phys. Med. Rehabil*. 2000; 81(7):910–5. 10896003 10.1053/apmr.2000.5615

[77] Watanabe K Kouzaki M Moritani T. Relationship between regional neuromuscular regulation within human rectus femoris muscle and lower extremity kinematics during gait in elderly men. *J. Electromyogr. Kinesiol*. 2018; 41:103–8. 29870932 10.1016/j.jelekin.2018.05.011

[78] Watanabe K. Relationship between toe clearance strategy and regional regulation of rectus femoris muscle during swing phase in prolonged walking in young and older adults. *Front. Physiol*. 2018; 9:1274. 30237768 10.3389/fphys.2018.01274 PMC6136235

[79] Dos Anjos FV Pinto TP Gazzoni M Vieira TM. The spatial distribution of ankle muscles activity discriminates aged from young subjects during standing. *Front Hum Neurosci*. 2017; 11:190. 28469567 10.3389/fnhum.2017.00190 PMC5395606

[80] Watanabe K Kouzaki M Moritani T. Effect of aging on region-specific functional role and muscle geometry along human rectus femoris muscle. *Muscle Nerve*. 2017; 56:982–6. 28044357 10.1002/mus.25556

[81] Miokovic T Armbrecht G Felsenberg D Belavy DL. Heterogeneous atrophy occurs within individual lower limb muscles during 60 days of bed rest. *J. Appl. Physiol (1985)*. 2012; 113(10):1545–59. 22984243 10.1152/japplphysiol.00611.2012

[82] Di Nardo F Fioretti S. Statistical analysis of surface electromyographic signal for the assessment of rectus femoris modalities of activation during gait. *J. Electromyogr. Kinesiol*. 2013; 23(1):56–61. 22841481 10.1016/j.jelekin.2012.06.011

[83] Winter DA Yack HJ. EMG profiles during normal human walking: stride-to-stride and inter-subject variability. *Electroencephalogr. Clin. Neurophysiol*. 1987; 67(5):402–11. 2444408 10.1016/0013-4694(87)90003-4

[84] Yang JF Winter DA. Surface EMG profiles during different walking cadences in humans. *Electroencephalogr. Clin. Neurophysiol*. 1985; 60(6):485–91. 2408847 10.1016/0013-4694(85)91108-3

[85] de Souza LM da Fonseca DB Cabral HD de Oliveira LF Vieira TM. Is myoelectric activity distributed equally within the rectus femoris muscle during loaded, squat exercises? *J. Electromyogr. Kinesiol*. 2017; 33:10–9. 28110043 10.1016/j.jelekin.2017.01.003

[86] Watanabe K. Effect of taping and its conditions on electromyographic responses of knee extensor muscles. *Hum. Mov. Sci*. 2019; 63:148–55. 30553140 10.1016/j.humov.2018.12.003

[87] Vieira TM Botter A. The accurate assessment of muscle excitation requires the detection of multiple surface electromyograms. *Exerc. Sport Sci. Rev*. 2020; 49:23–34. 10.1249/JES.0000000000000240 33044329

[88] Gallina A Ritzel CH Merletti R Vieira TM. Do surface electromyograms provide physiological estimates of conduction velocity from the medial gastrocnemius muscle? *J. Electromyogr. Kinesiol*. 2013; 23(2):319–25. 23265664 10.1016/j.jelekin.2012.11.007

